# A Digital Twin-Based Operation Status Monitoring System for Port Cranes

**DOI:** 10.3390/s22093216

**Published:** 2022-04-22

**Authors:** Yong Zhou, Zhengkang Fu, Jie Zhang, Wenfeng Li, Chaoyang Gao

**Affiliations:** 1 School of Transportation and Logistics Engineering, Wuhan University of Technology, Wuhan 430063, China; zhouyong@whut.edu.cn (Y.Z.); fzk_whut@whut.edu.cn (Z.F.); zhang_j@whut.edu.cn (J.Z.); 2 National NC System Engineering Research Center, Huazhong University of Science and Technology, Wuhan 430074, China; chaoyanggao@hust.edu.cn

**Keywords:** digital twin, port crane, monitoring system, anti-swing control, data interaction

## Abstract

To address the problems of the lack of an online data simulation test environment, the poor openness of data collection, and the low degree of data visualization in the online control process of port cranes, an operation state monitoring system framework for port cranes based on digital twins is proposed. In this framework, the digital twin port crane is used as the core, and the multi-sensor data acquisition method, OPC UA information model, and plug-in programming method are combined to realize multi-source heterogeneous virtual and real data fusion. The digital twin crane monitoring system based on this framework can fulfil the following functions: crane historical operation process reproduction, control program simulation testing, synchronous mapping simulation, and remote control. In order to verify the proposed method, a digital twin-based physical platform for monitoring a rail-mounted gantry crane (RMGC) was built, in which a virtual test of anti-swing control and a digital twin monitoring experiment were carried out. The results show that the virtual RMGC can test the control algorithm and map the movement process of the physical RMGC, and the crane operation monitoring system has high real-time performance and good visualization effect. In addition, the remote control of the software platform is accurate and effective.

## 1. Introduction

In the past 20 years, as a result of the trend of containerization of international trade, the throughput and tonnage of container ports have been gradually improved. Moreover, due to the influence of COVID-19, a large number of ships berthed at port to wait for unloading, thus requiring an improvement in port handling capacity [1]. Meanwhile, as a type of container handling equipment, port cranes have strong load periodicity, wide moving range, and large loads during operation, which indicate the need for higher requirements for its safety and reliability [2].

In order to improve the safety and reliability of cranes, many researches have studied the crane monitoring system [3,4]. These studies mainly focused on the aspects of information communication, system integration, and visualization. In terms of information communication, RFID, ZigBee, Wi-Fi, 5G and other technologies were applied in various crane monitoring systems. For example, Lee et al. [5] combined RFID and machine vision technology to provide crane operators with more detailed information about the working environment and objects to be transported during operation. Gao et al. [6] designed the swing angle measurement system of the crane based on a micro-electro mechanical system (MEMS) and sent the swing angle signal by ZigBee. Yang et al. [7] used multi-type sensors to collect the crane’s information, which was transmitted by Wi-Fi technology, and used MATLAB and ANSYS to analyze and visually display the stress and deformation of the crane. Shi et al. [8] used narrow band Internet of Things (NB-IoT) to transmit the data of the crane load and the motor current, which were uploaded to the cloud database for fault diagnosis and condition monitoring. Awad et al. [9] discussed the different applications of smart sensor networks on the cranes, and used an IoT-PLC control system to monitor the crane on the cloud. Li et al. [10] integrated 5G network and edge computing technology into the remote control of the crane, reducing the end-to-end control delay. In terms of system integration and visualization, digital mock-up technology has developed rapidly under the impetus of computer technology. For example, Price et al. [11] obtained crane working environment data through encoders, cameras, and laser scanning, so as to monitor the cranes in real time. They also established a graphic user interface (GUI) in Unity 3D to provide operators with three-dimensional visualization of crane status data, and used multi-sensor data for obstacle distance estimation and collision warning. Li and Liu [12] designed a data acquisition and transmission system based on a CAN bus, established a virtual model of tower crane in a parametric and modular manner, and used database data to drive the virtual crane movement. At the same time, they integrated the construction site data and virtual model to replace the camera monitoring system.

As a result of the development of a new generation of information and communication technologies, such as multi-sensor fusion technology, IoT, and cloud computing, digital twins have made considerable progress as a new technology that breaks the barrier between virtual and real data [13,14]. The digital twin is the mirror image of a physical entity in an information system through data and simulation software. It can be used to simulate, monitor, diagnose, and predict the formation process, state, and behavior of physical entities in the real environment, and provide more decision-making feedback for the actual system. The cyber-physical system (CPS) can realize the interactive linkage between the information virtual entity and the physical entity by constructing a closed-loop channel for data interaction between the information space and the physical space [15]. The digital twin and CPS have different characteristics in aspects of cyber-physical mapping, hierarchy, and core elements [16]. Simulation technology is a model technology that applies simulation hardware and software to reflect a system’s behavior or process with the help of numerical calculations and problem solving. Traditional simulation technology can only simulate the physical world offline, without the function of analysis and optimization [17]. Compared with traditional simulation, the digital twin relies on methods including simulation, measurement, and data analysis to perceive, diagnose, and predict the physical entity state, so as to optimize the physical entity and update the digital model to better reflect the current state of device. Compared with the hardware-in-the-loop simulation system, the digital twin adopts data for the whole system to drive the virtual system, and obtains more comprehensive information and feedback [18]. The digital twin acts in different application forms in different fields, but its model should generally conform to the basic model of real space–virtual space–linking mechanism proposed by Grieves [19]. Their common feature is the effective feedback provided to decision makers, physical systems, and information systems.

The digital twin has been utilized in the management and design of product lifecycles. Tao et al. [20] proposed a new method of product design, manufacture, and service driven by the digital twin and its corresponding application framework to solve the problem of serious data fragmentation between the product life cycle data and the various stages of the cycle in the virtual and real space. Liu et al. [21] established a digital twin model in the machining process based on biomimicry, simulating machining process from the three aspects of geometry, behavior, and environment to support machining decision making. Liu et al. [22] proposed a configuration, motion, control, and optimization (CMCO) architecture, and used the established digital twin model to conduct a hardware-in-the-loop simulation during the scheme design stage to detect and address potential design errors.

The digital twin is also used to improve monitoring systems. For example, Soares et al. [23] established a digital twin model of a sugarcane processing device, which has the ability to collect real-time data, such as the employee’s location and the device status for analysis, and provide twin information for the real-time decision-making process. Moi et al. [24] used the nonlinear finite element analysis method to simulate the stress process of the crane and established a virtual strain gauge to measure the strain data at the crane’s key positions. Lozano [25] developed a digital twin monitoring system for offshore cranes to provide real-time structural monitoring, and combined real-time sensing data and the digital twin model to realize early warning of the crane’s state. Cai et al. [26] fused the spindle vibration data and machining feature information into the digital twin three-axis vertical milling machine, so that it can better reflect the working state of the actual milling machine in the simulation machining process. Ding et al. [27] emphasized the interconnection and interoperability between physical and virtual workshops. They established a digital twin cyber-physical production system (DT-CPPS), which transformed the real-time data of the workshops into engineering information for the decision-making level, and improved the decision-making ability of the intelligent manufacturing system. Liu et al. [28] proposed a hoisting safety risk management framework based on a digital twin model, which realized the real-time perception and virtual–real interaction of multi-source information during the hoisting process of large-scale prefabricated buildings.

Combined with the application of the digital twin in other fields, it can be found that most of the existing crane monitoring system research has the following main problems:

1. Generally, only the data chart and the camera are used to reflect the equipment status. Firstly, due to the limited number of cameras, it is impossible to obtain a comprehensive view of the device. Secondly, it is difficult to understand the overall status of the equipment with limited measurement data. Thirdly, there is a lack of simple and effective means of human–machine interaction.
2. In terms of data collection and interaction, only data transmission methods are generally considered. However, when using hardware devices from different manufacturers, different software drivers need to be redesigned according to the hardware type to collect data, which leads to the low degree of openness of data collection.
3. The actual operating data and theoretical model data are not fused, resulting in a lack of online data simulation and analysis capabilities and a long prototype system development cycle.

Therefore, this paper proposes a port crane monitoring system framework based on the digital twin to support crane control algorithm testing and online monitoring of its operation process. Aiming at the problem (1), the digital twin crane is constructed from the aspects of geometry, underactuated motion, and anti-swing control. Combined with the data collected by various sensors, the status monitoring interface, and the digital twin model, the current status of the crane can be displayed from multiple viewpoints. In view of problem (2), this paper comprehensively considers the characteristics of various hardware products and designs a data interaction method combined with the information model of OPC UA, which can interact with various data across platforms, with low delay and scalability. In response to problem (3), multi-source data can be collected by multi-sensing methods, and various types of data are exchanged with information models to drive digital twin port cranes online. In addition, through in-depth fusion of virtual and real data, various control algorithms are verified online, reducing algorithm development time. Compared with the existing crane monitoring system, the monitoring system mentioned in this paper has the advantages of simple data collection and high platform openness, and can integrate virtual and real data for control algorithm testing, which improves the automation and intelligence level of port cranes.

## 2. Port Crane Monitoring System Framework Based on the Digital Twin

**Figure 1:**
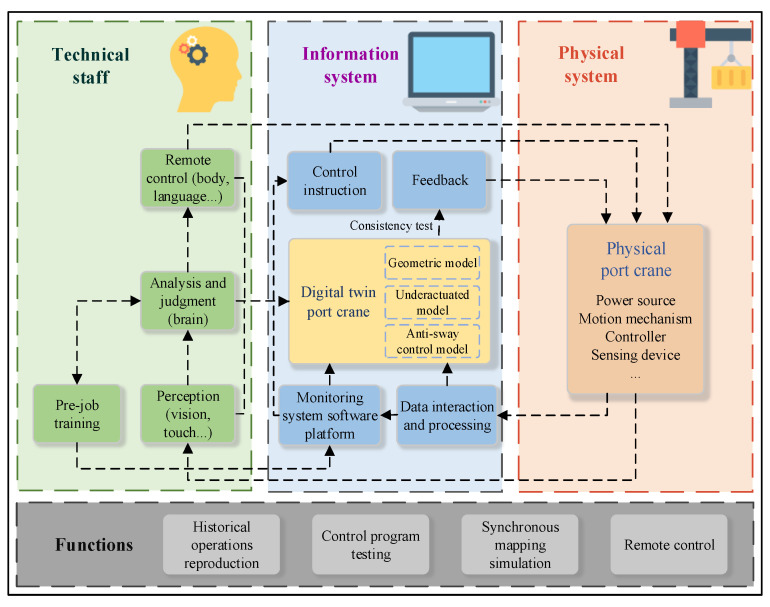
Framework of port crane monitoring system based on the digital twin.

The composition framework of the monitoring system proposed in this paper is shown in Figure 1. In the physical system, the multi-type sensors collect status data of the crane and surrounding environment data, and upload the data to the monitoring system software platform via industrial ethernet to complete the data collection and interaction. The crane control system receives the scheduling commands from the software platform and transmits the commands to each terminal actuator to complete the operation task of the crane. In the information system, through the establishment of a digital twin crane model, the control algorithm can be tested and optimized in advance. During the operation of the crane, the state of the crane is synchronously mapped to the virtual model with real-time data, and the early warning information is fed back to the physical system in time, which can improve the operation safety of the crane. The shape and size of the geometric model are built based on the physical model, and the consistency of the virtual and real motion attributes is ensured through the variables and actions bound by the online data-association model (data-constraint), so as to realize the ‘twin’ in vision and kinematics. The underactuated model describes the dynamic properties of the hoisting system, and the anti-swing control model optimizes the operation commands to reduce the residual swing angle of the lifting load. As participants, technical staff can assemble the crane operation knowledge into a knowledge base to provide data support for big data analysis, and remotely control the crane under necessary conditions according to operation experience. In addition, the software platform of the monitoring system can be used to operate the digital twin crane for pre-job operation training.

When technical staff interacts with the information system, not only can the data chart on the monitoring system software platform be used to view the current crane operation data, but the equipment’s running posture can also be accurately observed through the digital twin crane that is running synchronously. In addition, by using the software platform of the monitoring system, the digital twin crane can be controlled virtually to test the control algorithm, and the crane operation training can be carried out using a suitable good human–machine interface.

The functions in the framework mainly include reproduction of historical operations, control program testing, synchronous mapping simulation, and remote control. reproduction of historical operations means that the digital twin crane performs simulated movements according to the historical data of the database, for managers to analyze emergency handling methods in historical conditions, and operator training. The control program test uses the software platform to send control instructions to drive the digital twin crane, combined with the designed control model to virtually test the control algorithm. A synchronous mapping simulation drives the digital twin crane through actual crane data, continuously updates and maps the movement trajectory of the physical crane, and visually displays the movement status of the crane. Remote control is used to issue control instructions through the software platform to remotely operate the physical crane under specific circumstances.

## 3. Digital Twin Modeling of the Crane System

### 3.1. Real-Time Monitoring Physical Platform of Crane Based on Multi-Sensors

**Figure 2:**
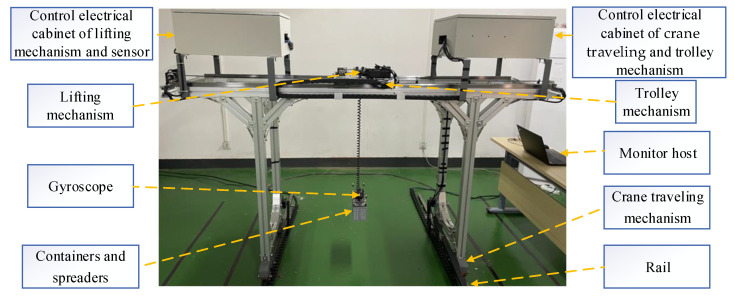
Photo of the miniature RMGC physical platform.

To verify the feasibility of the framework proposed in this paper, a miniature physical platform for a rail-mounted gantry crane (RMGC), which is commonly used in ports, was built, as shown in Figure 2. The controller used in the physical platform is an HPC-200 industrial personal computer (IPC) produced by Huazhong CNC Co., Ltd. (Wuhan, China), with a Baytrail 3815 CPU, 1G memory, and supporting EtherCAT bus. The control program compilation environment is the IEC61131-3 programming system HPAC developed by the iMC team of Huazhong University of Science and Technology. The software is a set of a graphical integrated development environment, and the RMGC control system program developed by it conforms to the IEC61131-3 standard.

**Figure 3:**
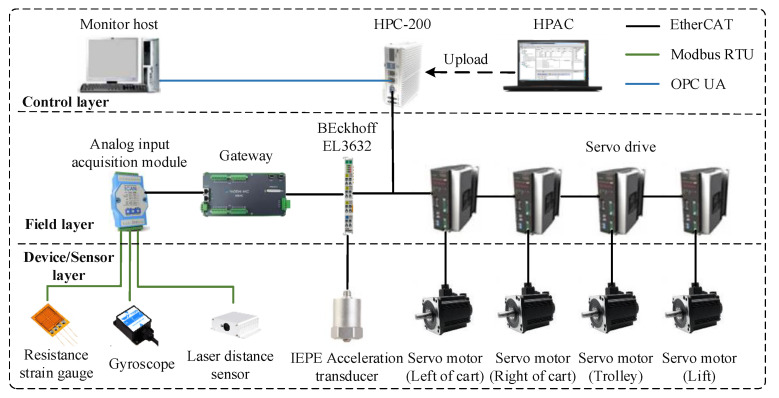
System architecture diagram of the physical platform.

As shown in Figure 3, the system architecture of the multi-sensor-based RMGC real-time monitoring physical platform consists of a multi-sensor acquisition module (including laser ranging sensors, gyroscopes, strain gauges, vibration sensors), an RMGC controller, monitoring host computer, software, etc. According to the different types of sensor communication interfaces and communication protocols, different acquisition programs are developed using the HPAC programming system and uploaded to the IPC to collect various sensor data. Then, the collected data preprocessed by the built-in communication script of the HPC-200 are sent to the monitoring host computer according to the communication protocol. At the same time, the IPC receives the remote operation instructions issued by the technical staff to control the movement of the crane.

### 3.2. Digital Twin RMGC Model

#### 3.2.1. Geometric Model

**Figure 4:**
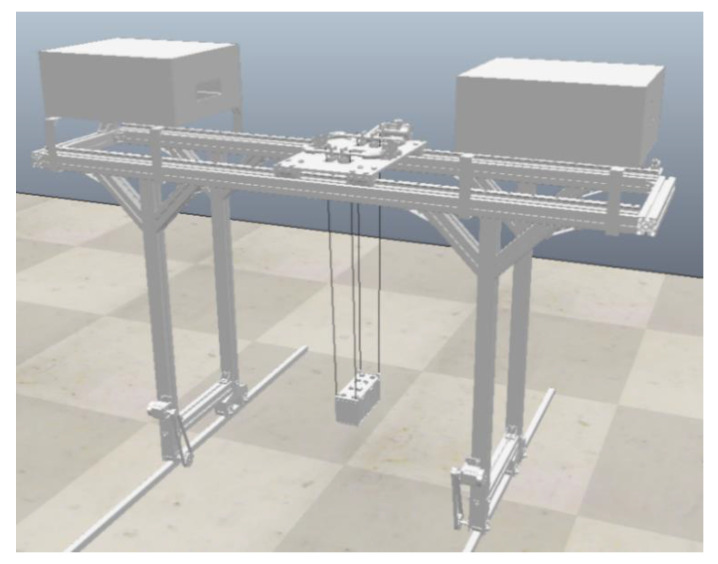
Three-dimensional model of the RMGC device based on CoppeliaSim (V-REP).

In the field of automation system simulation, building virtual scenes and virtual prototypes based on CoppeliaSim (V-REP) is a common research method [29]. CoppeliaSim (V-REP) is a powerful 3D integrated development environment, with a distributed control architecture, multiple optional physics engines, and support for multi-language script control. In this paper, the three-dimensional model of the RMGC device is established using CoppeliaSim (V-REP), as shown in Figure 4.

During the virtual simulation process, there are connections between the various mechanisms of the RMGC, between the RMGC and the container, and between the containers. This relationship can be described in the Python language using the script function of CoppeliaSim (V-REP). The motion of the RMGC model can be decomposed into the movement of the crane traveling mechanism, the trolley mechanism, and the lifting mechanism. Using the script function in CoppeliaSim (V-REP), we can also realize the data-driven virtual object and complete the mapping from the motion attributes of physical entities to virtual entities. In addition, the API function sim.checkDistance (clientID, Entity1, Entity2) in CoppeliaSim (V-REP) is used to calculate the relative distance between two objects for collision detection. When the distance is lower than a certain threshold, a warning message is sent in time to improve the security of the control algorithm.

#### 3.2.2. Dynamic Model of Load Swing System

**Figure 5:**
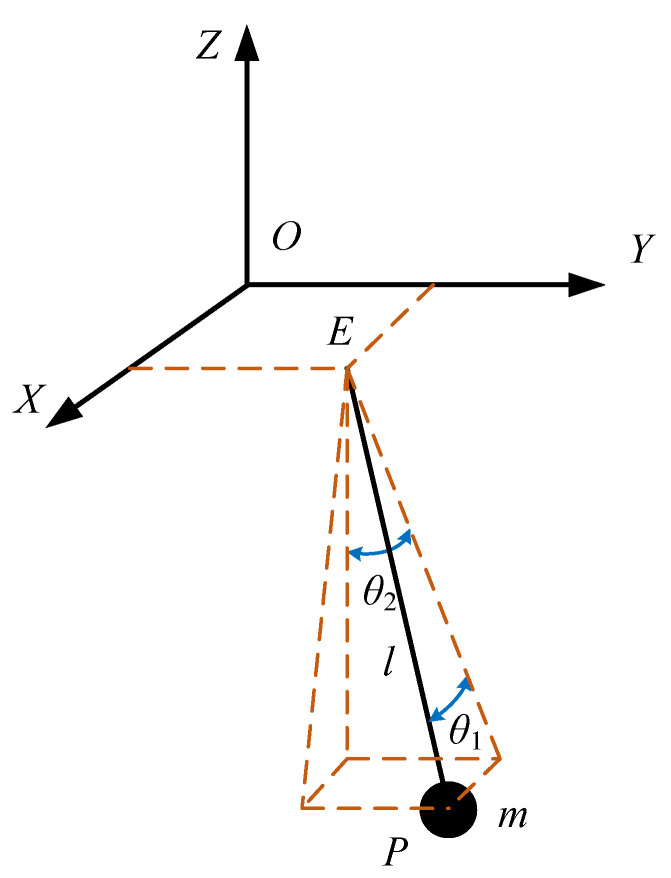
Schematic diagram of the load swing dynamic model.

The number of independent control variables of load motion in the RMGC is less than the number of system degrees of freedom, which is typical of an underactuated system [30]. The load swing system is simplified as a suspension point and a mass point connected by a wire rope, and the rectangular coordinate system *O-XYZ* is established in the load movement space to obtain the load swing dynamic model of the RMGC, as shown in Figure 5.

In this figure, the origin *O* of the coordinate system is the position of the motion starting point of the horizontal plane where the suspension point is located, the point *E* (*x_E_*, *y_E_*, 0) is the current position of the suspension point, and the point *P* (*x_P_*, *y_P_*, *z_P_*) is the position of the load center of mass; when the load rises or falls, point *P* is moves in the direction of *EP*, and *l* is the length of the wire rope *E*; *θ*_1_ is the included angle between *EP* and its projection on the *YOZ* plane, and *θ*_2_ is the included angle between the projection of *EP* on the *YOZ* plane and the vertical direction; *m* is the mass of the load.

It can be seen from the dynamic model that when the crane moves, point *E* moves in the direction parallel to the *OX* axis, and, when the trolley moves, point *E* moves in the direction parallel to the *OY* axis; then, the displacements of the crane and trolley are *x_E_* and *y_E_*, respectively. Thus, the load swings in three-dimensional space with the movement of the crane and trolley, and the change in the length of the wire rope.

Taking *x_E_*, *y_E_*, *θ*_1_, *θ*_2_, and *l* as the generalized coordinates, in terms of the geometric relationship, the coordinates of point *P* of the load can be obtained as follows:

(1) $$\left\{ \begin{array}{l} x_{p}=x_{E}+l\sin\theta_{1}\\ y_{p}=y_{E}+l\sin\theta_{2}\cos\theta_{1}\\ z_{p}=-l\cos\theta_{1}\cos\theta_{2} \end{array}\right.$$

According to the Lagrange equation, the dynamic equation of the load swing system can be obtained, as shown in Equation (2).

(2) $$\frac{d}{dt}(\frac{\partial L}{\partial {\dot{q}}_{i}})-\frac{\partial L}{\partial q_{i}}=F_{i}$$

where *L* is the Lagrange function; *q_i_* is the generalized coordinate, in which *i* is the number of complete constraint equations, while *q*_1_ = *x_E_*, *q*_2_ = *y_E_*, *q*_3_ = *θ*_1_, *q*_4_ = *θ*_2_, *q*_5_ = *l*; and *F_i_* is the generalized force in terms of the generalized coordinate system. When the swing angle of the load is small, the simplified condition of Equation (3) can be obtained, and then the dynamic equations corresponding to *θ*_1_ and *θ*_2_ can be simplified to Equation (4).

(3) $$\left\{ \begin{array}{l} \sin\theta_{j}\approx \theta_{j}\\ \cos\theta_{j}\approx 1\\ {\dot{\theta }}_{j}\sin\theta_{k}\approx 0\\ {\ddot{\theta }}_{j}\sin\theta_{k}\approx 0\\ \sin\theta_{j}\sin\theta_{k}\approx 0 \end{array}\right.j,k=1,2$$

(4) $$\left\{ \begin{array}{l} \overset{..}{\theta_{1}}+2\frac{\dot{l}\dot{\theta_{1}}}{l}+\frac{g\theta_{1}}{l}+\frac{\overset{..}{x_{E}}}{l}=0\\ \overset{..}{\theta_{2}}+2\frac{\dot{l}\dot{\theta_{2}}}{l}+\frac{g\theta_{2}}{l}+\frac{\overset{..}{y_{E}}}{l}=0 \end{array}\right.$$

It can be seen from Equation (4) that the swing angles of the load are related to the running accelerations of the crane and the trolley, wire rope length, and lifting speed, whereas the motion of the crane and trolley has no coupling effect on the swing angles of the load. Therefore, *θ*_1_ and *θ*_2_ can be solved separately. During the operation of the digital twin RMGC, the swing angle of the load can be obtained by solving Equation (4) with the fourth-order Runge–Kutta method, and then the position of the load can be continuously updated by Equation (1) to describe the kinematic and dynamic properties of the load. Combined with the anti-swing control model below, the control program can be tested in a virtual scene.

#### 3.2.3. Anti-Swing Control Model

As an open-loop control method, the input shaping convolutes the original input signal of the control system with the pulse signal generated in the shaper to obtain the shaping signal, which acts as a new input to the system to control the residual vibration [31].

Considering that the hoisting mechanism of the RMGC is generally stopped when the crane and the trolley are running to ensure safety—that is, the change in the rope length is not considered during the operation—the traditional zero vibration (ZV) shaper is adopted in this paper. The ZV shaper has the advantages of short adjustment time and simple design. The acceleration of the crane or trolley is shaped by the ZV shaper, and the swing of the load will be canceled by the action of two pulse signals.

The ZV input shaper can be designed to contain two pulses, which meets the constraint condition Equation (5), and the control parameters can be obtained by solving Equation (6) [32].

(5) $$\left\{ \begin{array}{l} V(\omega_{n},\xi )=0\\ A_{1}+A_{2}=1\\ t_{1}=0 \end{array}\right.$$

(6) $$\left\{ \begin{array}{l} A_{1}=\frac{1}{1+K};\;A_{2}=\frac{K}{1+K}\\ t_{1}=0;\;t_{2}=\frac{\pi }{\omega_{n}\sqrt{1-\xi^{2}}}\\ K=e^{-\xi \pi /\sqrt{1-\xi^{2}}} \end{array}\right.$$

where *V*(*ω_n_*, *ξ*) is the ratio of the residual vibration amplitude of the system with shaping to that of the system without shaping.

### 3.3. Virtual and Real Data Interaction Based on OPC UA

**Figure 6:**
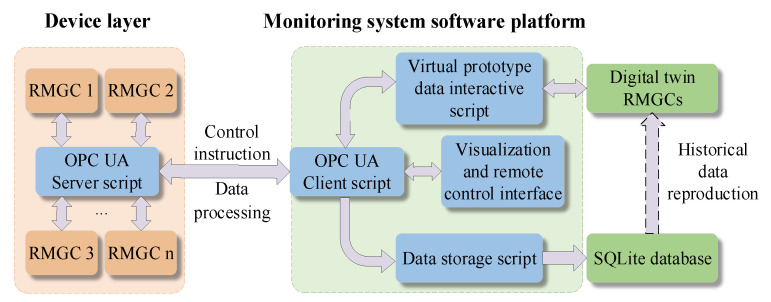
Schematic diagram of data interaction based on OPC UA.

In system-level data interaction, developers usually encounter the problem of low data collection openness by designing different drivers to collect status data of devices from different hardware suppliers. For the monitoring system of the RMGC, most of the data are captured in real time and the source is complex. In addition, data interaction between the digital twin RMGC and physical equipment is required, and then the interaction and integration between virtual and real data becomes more difficult. OPC UA has a low average communication delay. Ferrari et al. [33] verified that the average cost of data transmission from a Siemens S7 1500 Controller to an IBM Bluemix Platform is 70 ms. Lee et al. [34] used OPC UA to establish a real-time data transmission and reception connection between two-wheeled robots, with an average delay time of 20 ms. In addition, Mühlbauer et al. [35] verified that OPC UA has excellent scalability, which is determined by the information model of OPC UA and its client–server response mechanism. In this paper, the open OPC UA protocol is used for data interaction [36,37,38] between the monitoring host and the controller to break through the communication barriers between the virtual and the real, or between different hardware devices. The data interaction in the whole monitoring process is shown in Figure 6.

The OPC UA server script embedded in the IPC reads the data required by the monitoring system in real time, and conducts preprocessing steps on the data after acquiring it, such as filtering, unifying the type, and structuring after removing the unit, and then transmits it to the software platform via ethernet. After the OPC UA client script in the monitoring computer obtains the data uploaded by the server and decodes the data, the computer CPU starts multi-threading, and sends the data to the digital twin RMGC through the virtual and real interactive data script for synchronous mapping simulation, and to the SQLite database through the data storage script for data storage. The OPC UA client script can send the operator’s control instructions to the server, and then convert them into control codes through the control program to control the equipment. In addition, to facilitate offline reproduction of historical working conditions, the digital twin RMGC should have the ability to extract historical data from the SQLite database for offline simulation.

## 4. Verification of the Scheme

### 4.1. Monitoring Process of Digital Twin-Driven RMGC

**Figure 7:**
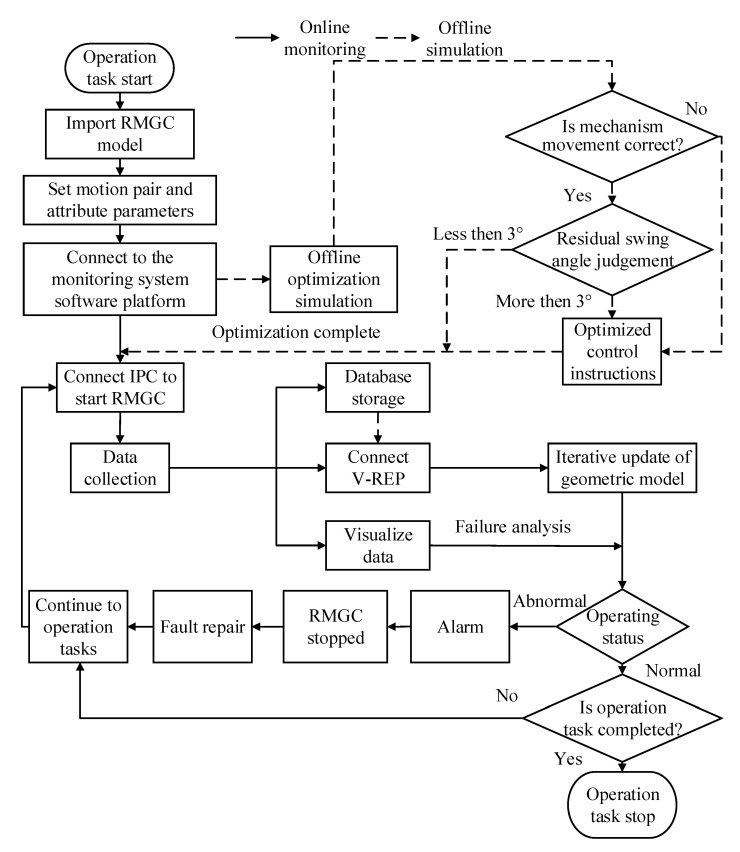
Flowchart of monitoring and control of the digital twin-driven RMGC.

The digital twin-driven RMGC monitoring system takes the digital twin RMGC as the core, and combines various functional applications to provide a 3D visualization scene and control algorithm test environment for the safe and efficient operation of the RMGC. Thus truly and synchronously reflects the operation status of the physical RMGC, and can perform fault diagnosis based on monitoring data. On this basis, the remote control function can also be used to send control commands to the control system of the physical RMGC to solve the abnormal problem. The real-time monitoring process is shown in Figure 7.

In the monitoring process, the environmental parameters and the motion properties of each mechanism of the geometric model of the RMGC established by CoppeliaSim (V-REP) should be set first. Then, the control command parameters obtained using the anti-swing control model are tested and optimized in the virtual scene. After the optimization test is completed, the software platform sends the control command parameters to IPC via industrial ethernet with OPC UA as the data link layer, and starts to control the physical RMGC for operation. During operation, the multi-sensor acquisition and interaction system collects the real-time position, speed, stress, vibration, sling swing angle, and other multi-source heterogeneous data of the RMGC for analysis. Combined with CoppeliaSim (V-REP) synchronous simulation, the state of the RMGC can be monitored from multiple angles. If an abnormal state occurs, the software platform will display alarm information and send a signal to suspend the operation to avoid possible injury to the RMGC or operators. After troubleshooting the corresponding exceptions, the operation task can be continued until the task is completed.

During real-time monitoring, the data acquisition system uploads the data to the software platform, which stores and visualizes the data separately and connects to CoppeliaSim (V-REP) for synchronous simulation. The stored data can be used to reproduce the historical operation process for further data analysis or conduct pre-job safety training. Among the data, the visual monitoring data in the form of graphs or tables can be combined with the status prompts after data analysis to jointly reflect the current operating status of the RMGC, so that users can view and stop dangerous operations in time. At the same time, the geometric model in CoppeliaSim (V-REP) is simulated iteratively according to the real-time operation data of each component, so as to realize the 3D visual monitoring effect of the RMGC operation.

### 4.2. OPC UA Data Interaction Delay Analysis

**Figure 8:**
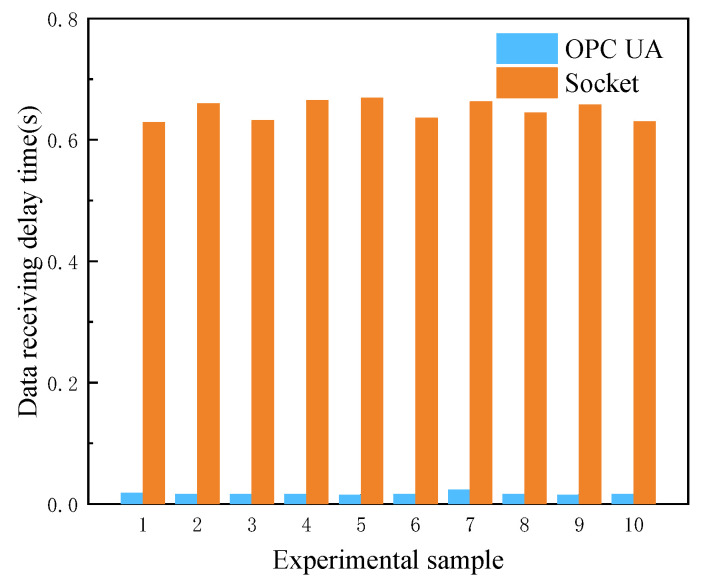
Communication time delay comparison.

On the client of the software platform, OPC UA and Socket protocols are used to deliver and receive control signals, respectively, so as to verify the advantages of OPC UA communication protocol in data acquisition and processing. Here, the motion control of the crane is taken as an example. Ten groups of experiments on communication time delay were carried out for comparison, starting at the time when the control signal was sent and ending at the time when the truck position data changed in the data packet after data parsing. The experimental results are shown in Figure 8.

It can be seen from Figure 8 that the average delay time of OPC UA in receiving data is 0.01670 s, whereas the average delay time of Socket in receiving data is 0.6487 s. This indicates that OPC UA has the characteristic of low delay in the data interaction between the monitoring host and controller designed in this paper.

### 4.3. Virtual–Real Consistency Test

**Figure 9:**
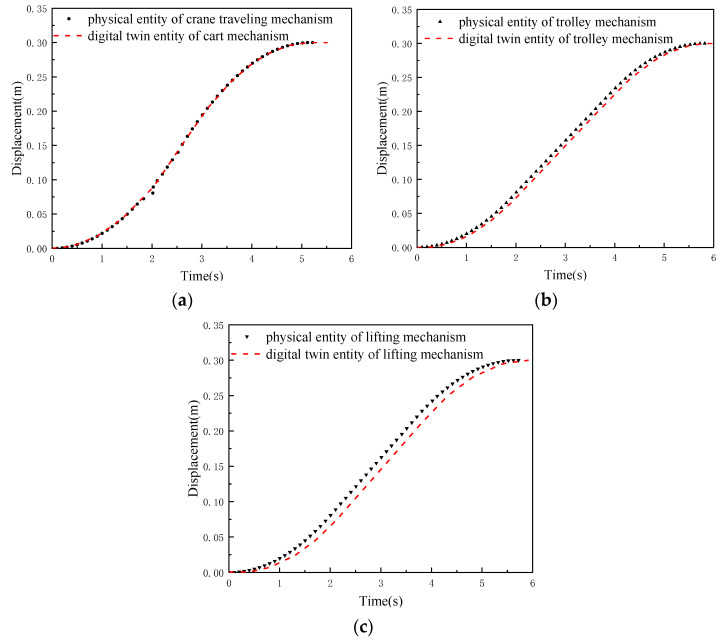
Experimental results of virtual and real displacement consistency: (**a**) crane traveling mechanism; (**b**) trolley mechanism; (**c**) hoisting mechanism.

In order to verify the accuracy and the reliability of the data-constrained driving mode, the crane traveling mechanism, the trolley mechanism, and the lifting mechanism were commanded to move 0.3 m, respectively. The encoder on each servo motor is used to record the current position of each mechanism of the physical RMGC, and the collected data are used to drive the movement of the digital twin RMGC. At the same time, the function sim.getObjectPosition (Entity1, Entity2) in CoppeliaSim (V-REP) is used to obtain the position of the digital twin RMGC. The comparison of the displacement results of each mechanism is shown in Figure 9.

It can be seen from Figure 9 that the motion attributes of the virtual and physical entities are basically identical. Since the virtual–real data interaction and the CoppeliaSim (V-REP) simulation calculation take a period of time, the movement in the virtual entity occurs later than that in the physical entity. The delay time is closely related to the performance of the computer and the design of the data interaction program.

### 4.4. Virtual Test of Anti-Swing Control for Digital Twin-Driven RMGC

Generally, the test of the control algorithm requires the use of a standard controller to directly control the physical entity, which requires good testing conditions and large initial investment. As a result of the introduction of the digital twin, the developer can test the control algorithm in the virtual environment in the early stage of algorithm development, and judge the control effect beforehand. In the middle stage of development, the actual controller can be used to control the digital twin entity to check the control method, thus reducing the construction cost of the prototype. In the later stage of development, the data obtained by the digital twin can be used to reflect the running state of the actual control system. It should be noted that, considering the real-time performance and security of the control system, the digital twin control system in this paper does not directly participate in the real-time control of the actual controller, but provides information for decision makers, and then feeds information back to the actual controller, which is indirect data feedback.

In order to verify the practicability and feasibility of the port crane monitoring system framework based on the digital twin, experiments were carried out according to the designed RMGC monitoring process. The first stage of the process was the virtual optimization test of the control program by the digital twin RMGC. Here, the trolley operation control was tested. The operation conditions were as follows: the trolley operation displacement was 1.0 m, the maximum operation speed was 0.2 m/s, and three groups of experiments were carried out with accelerations of 0.4 m/s^2^ (condition 1), 0.8 m/s^2^ (condition 2) and 1.0 m/s^2^ (condition 3).

**Figure 10:**
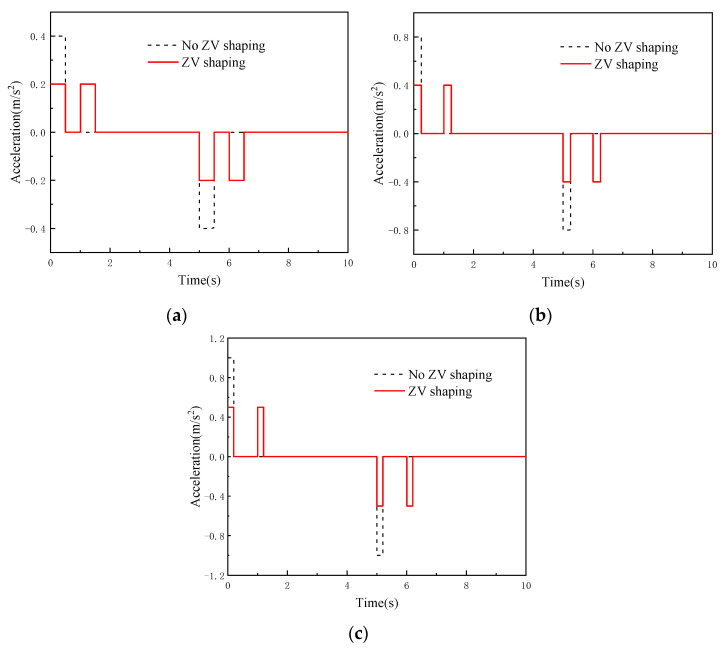
Acceleration curves of the trolley before and after shaping: (**a**) condition 1; (**b**) condition 2; (**c**) condition 3.

**Figure 11:**
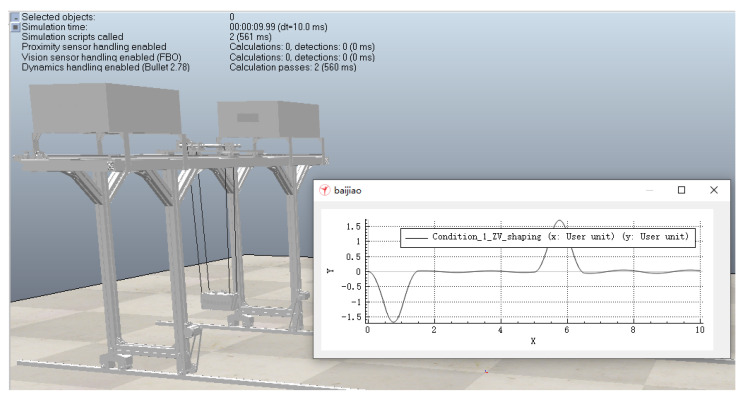
Digital twin RMGC for virtual testing.

**Figure 12:**
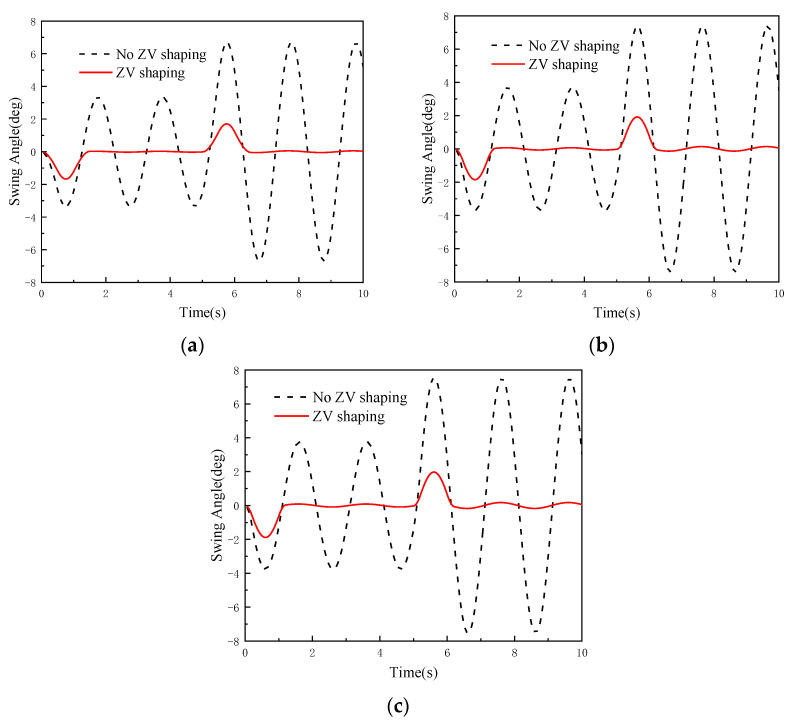
Comparison of swing angle curves obtained by simulation under different working conditions: (**a**) condition 1; (**b**) condition 2; (**c**) condition 3.

According to the anti-swing control model, the monitoring system software platform sends the control parameters under three working conditions to the digital twin RMGC, and then the digital twin RMGC shapes the acceleration of the trolley using the ZV method. The acceleration curves of the trolley before and after shaping are shown in Figure 10. The virtual test process of the digital twin RMGC is shown in Figure 11 and the obtained load swing angle curves are shown in Figure 12.

It can be seen from Figure 12 that after shaping, the maximum swing angles during operation under the three working conditions are reduced by 74.63%, 73.44%, and 73.05%, respectively, and the maximum residual swing angles are reduced by 99.2%, 98.16%, and 97.7%, respectively. This shows that the digital twin RMGC can simulate and test its operation control and visualize the operation process, laying a foundation for the actual operation control of the physical RMGC.

### 4.5. Online Monitoring Experiment of RMGC Based on Digital Twin

**Figure 13:**
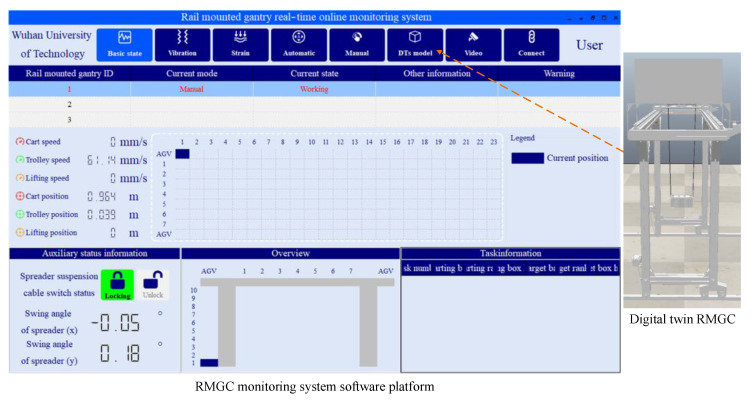
Monitoring system interface.

To further verify the applicability of the proposed monitoring system, the accuracy of the control program test, and the real-time performance of virtual real mapping, an on-line monitoring experimental study of virtual-real combination was carried out. The monitoring system software platform sends the control parameters obtained by simulation optimization to the physical RMGC to remotely control its movement. During the movement, the strain of the important structure of the RMGC, the vibration of the span structure, the running position and speed of each mechanism, and the swing angle of the spreader are measured. At the same time, using the above multi-source heterogeneous data interaction scheme, the digital twin RMGC is directly driven in the form of a data motion joint, and the physical RMGC is monitored online in combination with the mapping results of the digital twin RMGC and the monitoring data of the software platform. The online monitoring system interface is shown in Figure 13.

**Figure 14:**
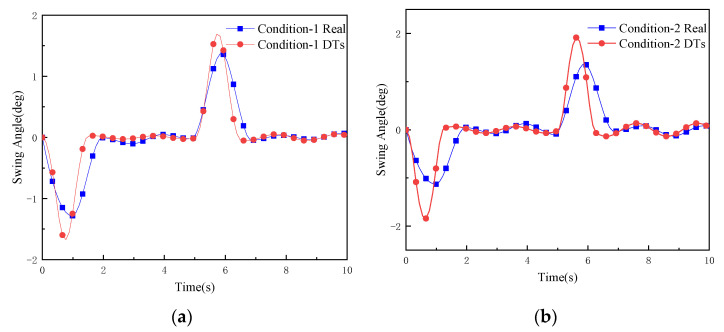

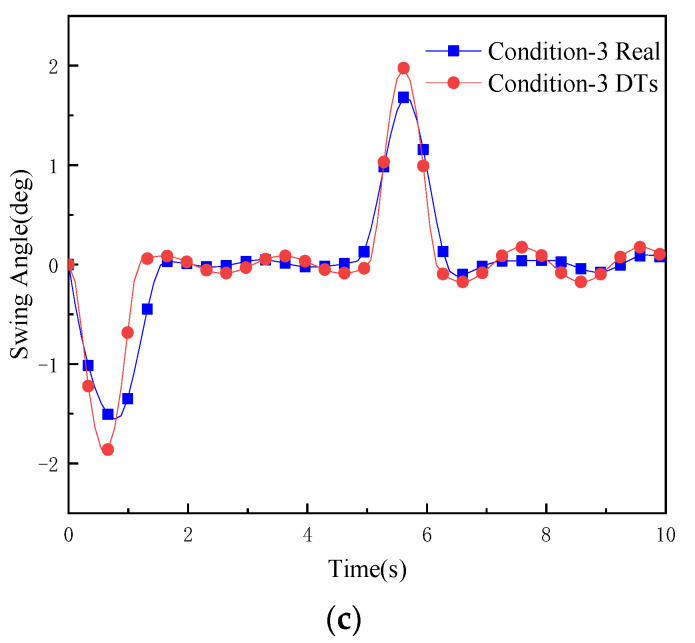
Comparison of load swing angles in Condition Real and Condition DTs under three working conditions: (**a**) condition 1; (**b**) condition 2; (**c**) condition 3.

To verify the effectiveness and accuracy of the virtual test of the digital twin-based anti-swing control, the real swing angle data (Condition Real) of the physical RMGC measured by the gyroscope under three working conditions were extracted and compared with the swing angle data obtained by the digital twin RMGC virtual test (Condition DTs) using the ZV shaping method. The results are shown in Figure 14.

As can be seen from Figure 14, under the three working conditions, the measured swing angle of the physical RMGC is basically consistent with that obtained from the virtual test, indicating that the digital twin RMGC can effectively test the control algorithm.

In addition, to determine the delay of data transmission, in this study, the time stamps carried by the data during 20 data transmissions were statistically compared, and the average time for data to complete one transmission in the system was calculated to be 0.11765 s, which can meet the transmission requirements of RMGC monitoring data.

## 5. Conclusions

In this paper, a digital twin-based port crane monitoring system framework is proposed, and the feasibility and effectiveness of the proposed framework are verified with the online monitoring of RMGC as an example. Compared with the existing monitoring system, the new monitoring system has a high system integration degree, which enriches the interactive means of the port crane monitoring process and the test environment of the control algorithm, and improves the openness of data collection and the degree of data visualization. In the case study of the monitoring system, a multi-sensor real-time monitoring physical platform of an RMGC was built based on the IEC61131-3 standard. Then, the digital twin model of the RMGC was built, including the geometric model of the RMGC based on CoppeliaSim (V-REP), the dynamic model of the load swing system, the anti-swing control model based on the input shaping method, and the virtual and real data interaction based on OPC UA. The monitoring system has the functions of real-time online monitoring, historical operation reproduction, motion control program testing, synchronous mapping simulation, and remote control. Combined with the multi-sensor acquisition and interaction system, it can realize the synchronous mapping of virtual and real models. The experimental results indicate that the monitoring system proposed in this paper has high real-time performance, and the digital twin RMGC can map the motion process of the physical RMGC and test the control algorithm. In addition, the model visualization effect is good, and the remote control of the software platform is accurate and effective.

In future work, OPC UA will be compared with other industrial protocols in terms of data transmission stability, delay and CPU operation, and storage consumption in the case of massive data interaction, so as to provide a reference for its application in different digital twin scenarios. In addition, although this study found that the proposed digital twin-based operation status monitoring system can improve the safety of the crane, the crane safety problem itself was not discussed in detail in the current paper. Thus, this can be the focus of the following research, including using the digital twin model to study the anti-collision features of the crane.

## Data Availability

Not applicable.
